# Closed suction drainage offers no more clinical benefit than non-drainage after primary total knee arthroplasty with the administration of tranexamic acid in Chinese patients

**DOI:** 10.1186/s42836-020-00035-7

**Published:** 2020-06-23

**Authors:** Dong Yang, Kaiyuan Liu, Lin Fan, Tianyang Xu, Guodong Li

**Affiliations:** Department of Orthopedics, Shanghai Tenth People’s Hospital, No. 301, Yanchang Road, Shanghai, 200072 People’s Republic of China

**Keywords:** Total knee arthroplasty, Drainage, Tranexamic acid, Retrospective

## Abstract

**Background:**

If closed suction drainage (CSD) should be used after primary total knee arthroplasty (TKA), remains controversial. The current study aimed to explore whether CSD offers more clinical benefit with the administration of tranexamic acid in Chinese patients.

**Methods:**

A retrospective study was conducted on 200 patients who had received TKA. One hundred patients were placed on drainage after TKA, whereas the rest of 100 patients were not. Multiple clinical parameters were dynamically monitored and compared between the two groups.

**Results:**

The length of the hospitalization days was significantly shorter in patients who did not receive CSD (6.0 ± 0.8 *vs*. 7.0 ± 0.8 days, *p* < 0.001). The patients in the drainage group had more Hb drop on the first postoperative day (14.82 ± 6.58 *vs*. 11.27 ± 5.71 g/L, *p* < 0.001). No statistically significant difference was observed in VAS score, ROM and thigh circumference at baseline during the follow-up between two groups.

**Conclusions:**

Additional drainage after primary TKA had no clinical benefit after the tranexamic acid had already been administered during the treatment.

## Introduction

TKA has been proved to be a successful and standardized procedure for reducing pain and restoring function in patients with end-stage knee osteoarthritis [1]. As a routine step for TKA, a CSD installed before the closure of joint capsule was believed to be able to prevent hematoma formation and decrease the incidence of wound-related complications [2]. However, recent studies showed the otherwise. Multiple clinical trials indicated that CSD offered no additional benefits after TKA [3–5]. In addition, many CSD-related side effects, including retrograde infection, increased the loss of blood, and prolonged hospital stay time, were reported [6].

Intraoperative use of tranexamic acid has resulted in the paradigm shift of blood management in modern total joint arthroplasty. It has been demonstrated to be an effective method to reduce blood loss and transfusion requirements due to its antifibrinolytic effect [7–9]. A question presents itself: Is CSD still clinically necessary after tranexamic acid is introduced? More investigations are warranted to answer this question.

The aim of this retrospective study was to investigate the benefit of CSD, as compared to no use of drainage, after primary TKA with the administration of tranexamic acid in Chinese patients. We empirically hypothesized that, compared to CSD, not to use drainage has the advantages of decreasing Hb loss and shortening the length of hospital stay. Moreover, we believe there are no differences in the incidence of complications and the benefit in knee function rehabilitation.

## Methods

### Patients and study design

**Fig. 1:**
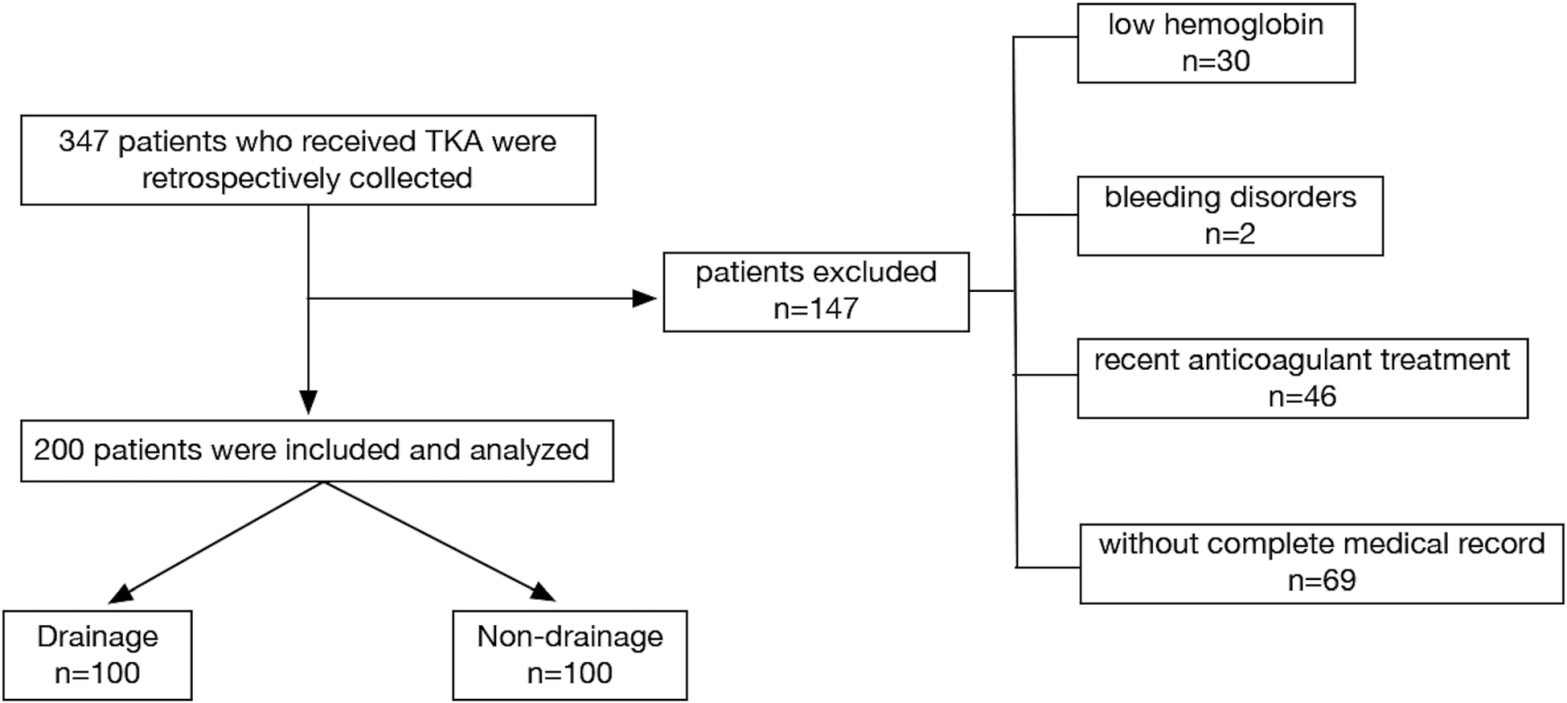
Study flow chart

In this retrospective study, 347 patients receiving or without receiving drainage after TKA were enrolled from January 2018 to January 2019 in Shanghai Tenth People’s Hospital, and, of them 147 patients were excluded. The exclusion criteria were as follows: (1) low hemoglobin level: Hb < 120 g/L in men and < 110 g/L in women; (2) patients with bleeding disorders, such as hemophilia, aplastic anemia; (3) patients who had been on anticoagulant treatment for 2 weeks; (4) patients whose medical records or testing results were not available. Finally, 100 patients receiving drainage and 100 patients without receiving drainage were included, respectively (Fig. 1).

### Surgical procedure

All patients received TKA under the same anesthetic protocol in combination with a single-shot femoral nerve blockade. On the premise of the tourniquet, a standard procedure involving a posterior cruciate-substituting cemented prosthesis (Genesis II, Smith & Nephew) was performed by a senior surgeon (Prof G. Li). A longitudinal midline incision and a mini midvastus approach were used. After removal of tibial and femoral osteophytes, intramedullary and extramedullary guides were placed to facilitate the cutting of femur and tibia, respectively. High-pressure pulsatile irrigation was then used to clean the bone surface and soft tissues. Before installation of the implants, 100 ml “cocktail” (a mixture of a single dose of morphine, ropivacaine, triamcinolone and 300 μL adrenaline, diluted by normal saline) was injected into soft tissues around the knee. Following the introduction of the components, the tourniquet was released immediately and the wound was then closed. In terms of if a drainage tube was placed, patients were divided into a CSD group and a non-CSD group. All patients were intra-articularly injected TXA after closure of the joint capsule, and the CSD was retained no more than 24 h after surgery.

### Evaluation of clinical parameters

#### Hemoglobin (Hb)

Patients’ Hb level was determined before and 1, 3,5 day(s) and 2 weeks after the operation. The drop of Hb from the baseline indirectly reflected the blood loss.

#### Pain

Visual Analog Scale (VAS) was employed to evaluate the knee pain perceived by patients. The pain was qualitatively rated on a 10-point scale, with no pain listed as 0, light pain as 1–3 point(s), moderate pain as 4–7 points and severe pain as 8–10 points. Individual patients were scored against VAS before and 1, 3, 5 day(s) and 6 months after operation. Postoperative VAS scores were the sum of the scores of the thigh pain, rest and active pain of the knee.

#### Range of motion (ROM)

ROM in flexion and extension of the operated knee was evaluated before and 1, 3, 5 day(s) and 6 months 6 after the operation.

#### Thigh circumference

The thigh circumference was used as an indicator of limb swelling. It was measured 10 cm proximal to the superior border of the patella. The dynamic change of the circumference reflected the ability of two groups in easing the instant stress and damage caused by the operation.

### Statistical analysis

All data were analyzed by using the Statistical Package for Social Science (SPSS) software (version 23.0 for Mac) and GraphPad Prism software (version 6 for Mac). Qualitative data were compared by using a Chi-square test. Numeric data were presented as mean ± standard deviation and *t*-test was used to evaluate the significance of differences between independent samples. *P* values in this article were two-sided and considered statistically significant when they were less than 0.05.

## Results

### Clinical features of the subjects

In this study, 100 patients were included in the CSD group and 100 in the non-CSD group, respectively. The median age was 75.1 years, ranging from 65 to 89. 158 patients (79.0%) were male, with 80 of them receiving drainage after primary TKA. As shown in Table 1, the ratios of age and gender had no statistically significant differences between the two groups. The mean body mass index (BMI) was 25.0 ± 3.21 kg/m^2^ and no statistical difference in BMI was found between the two groups. The time of straight-leg raising (SLR) was 22.98 ± 1.88 h in the drainage group whereas in the non-drainage group, the time was 23.00 ± 2.11 h and no significant difference in SLR was observed between two groups. Of note, the length of hospitalization was significantly shorter in patients who didn’t receive drainage than in the drainage group (6.0 ± 0.8 vs. 7.0 ± 0.8 days, *p* < 0.001).

**Table 1:** Clinical characteristics of included patients

Parameters	Drainage (*n* = 100)	Non-drainage (*n* = 100)	Total (*n* = 200)	*p* value
Age, year (range)	75 (65–89)	74.5 (67–84)	75.1 (65–89)	0.575
Gender				
Male	20 (20.0)	22 (22.0)	42 (21.0)	0.728
Female	80 (80.0)	78 (78.0)	158 (79.0)	
BMI (kg/m^2^)	25.25 ± 3.34	24.5 ± 2.38	25.0 ± 3.21	0.322
Hospitalization time (days)	7.0 ± 0.8	6.0 ± 0.8	6.5 ± 0.91	**< 0.001**
SLR (h)	22.98 ± 1.88	23.00 ± 2.11	22.99 ± 1.99	0.944

Data are shown as mean ± standard deviation or numbers (%)

*Abbreviations*: *BMI* body mass index, *SLR* straight-leg raising

### Effect of drainage and no drainage on various clinical parameters after TKA

#### Hemoglobin

**Fig. 2:**
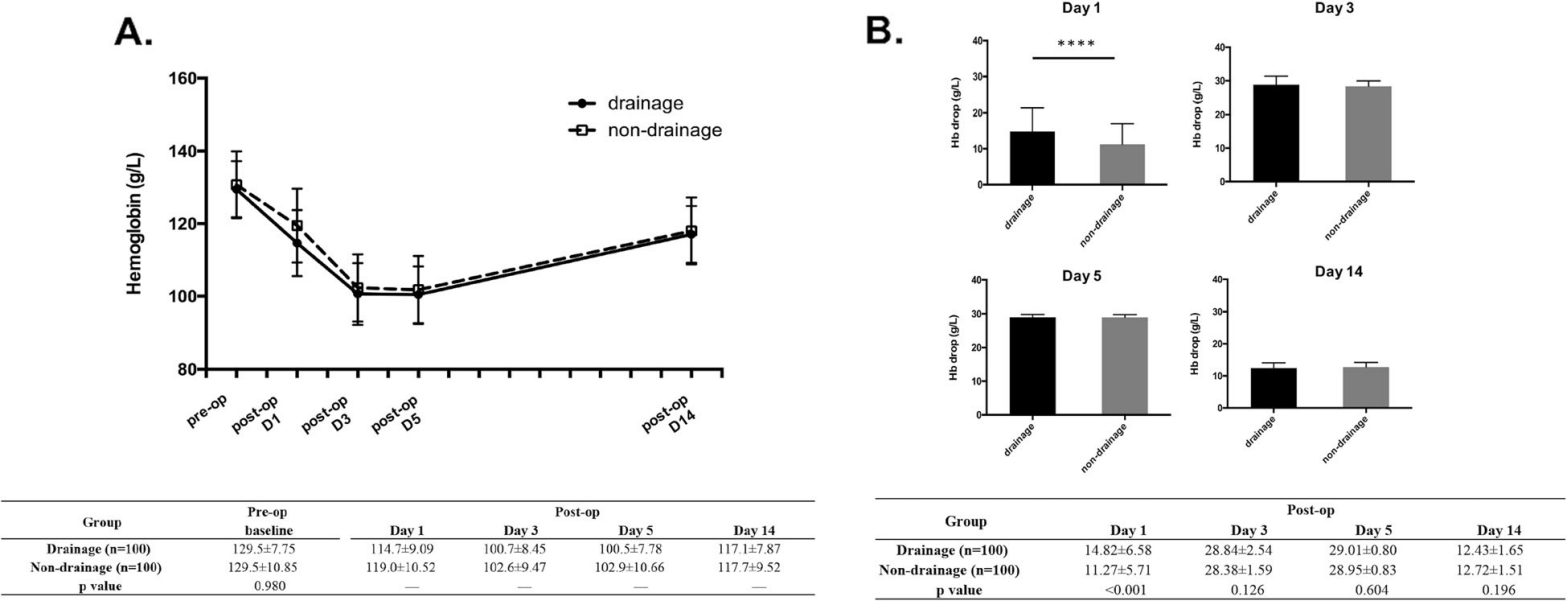
Effect of drainage on hemoglobin level after TKA. **a**. the dynamic change of Hb level; **b**. Hb drop at different time point compared with baseline level

There existed no significant difference in the baseline level of Hb between the two groups (*p* = 0.98). The hemoglobin in both groups dropped first and then rose, as shown in Fig. 2a. However, patients who received drainage after TKA suffered from more Hb drop than those without drainage on postoperative day 1 (14.82 ± 6.58 *vs*. 11.27 ± 5.71 g/L, *p* < 0.001) (Fig. 2b). Except for Day 1, the two groups showed no significantly differences in the drop of hemoglobin at other time points.

#### VAS score

**Fig. 3:**
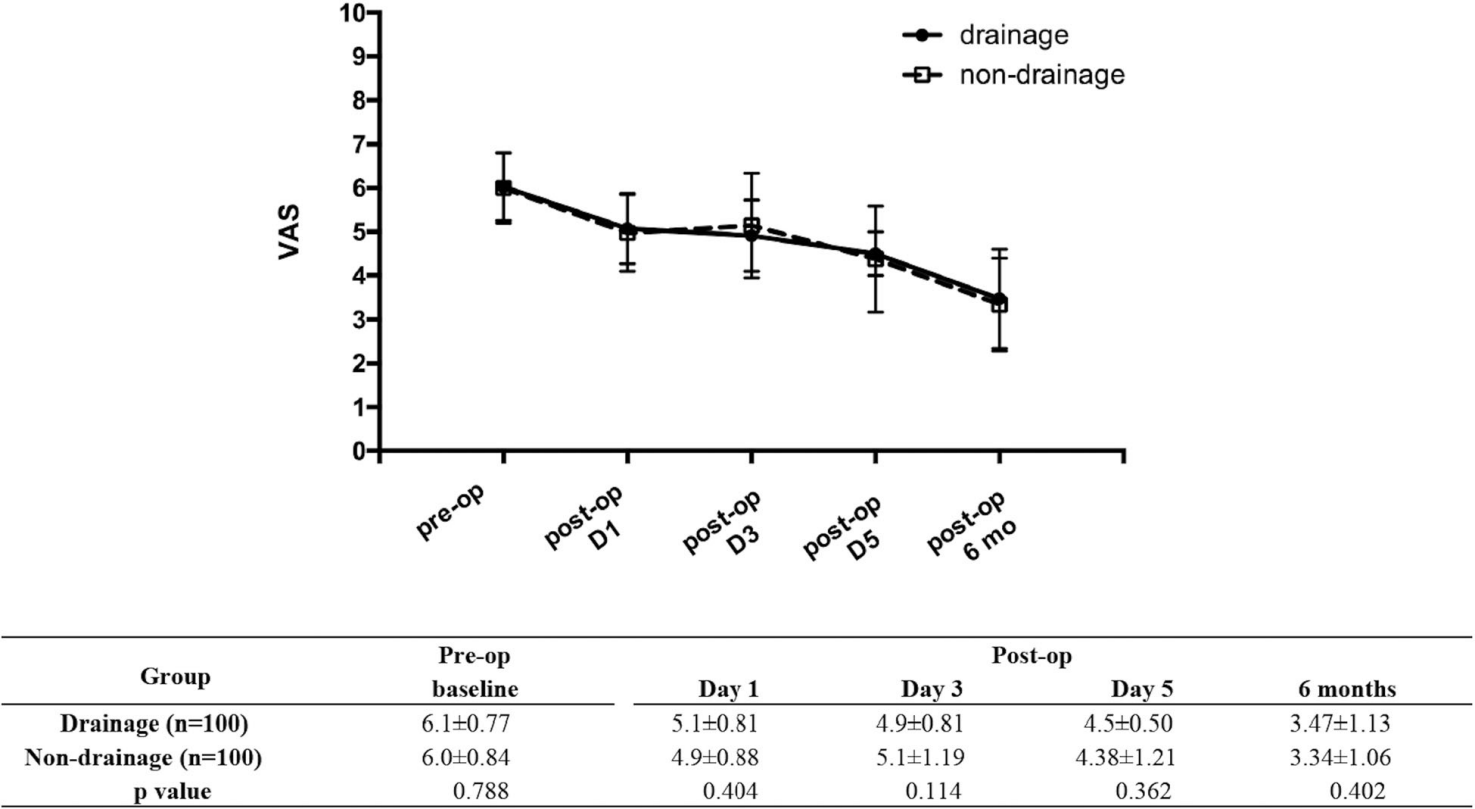
Effect of drainage on VAS score after TKA

Follow-up showed that pain diminished over time in both two groups. No statistically significant differences in VAS scores were found between the drainage group and non-drainage group 1, 3, 5 day(s) and 6 months after the operation (Fig. 3).

#### ROM

**Fig. 4:**
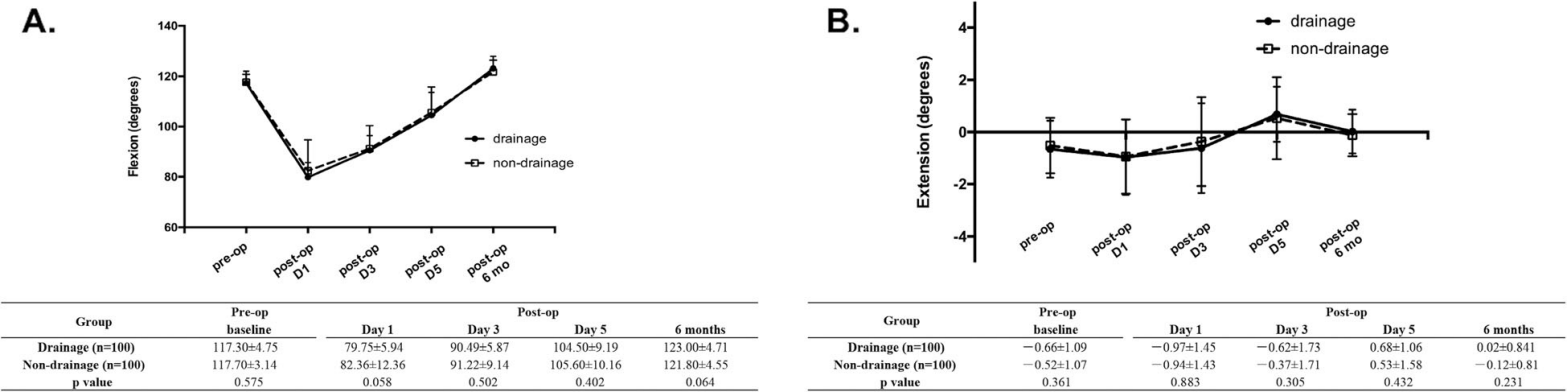
Effect of drainage on ROM after TKA. **a**. flexion degree; **b**. extension degree

In terms of ROM, no difference was observed between the two groups at baseline (flexion: *p* = 0.575; extension: *p* = 0.361). Interestingly, at Day1, patients who didn’t receive drainage had marginally more flexion than those who received drainage (79.75 ± 5.94 vs. 82.36 ± 12.36 °, *p* = 0.058). However, longer follow-up showed no differences in flexion or extension between the two groups (Fig. 4).

#### Thigh circumference

**Fig. 5:**
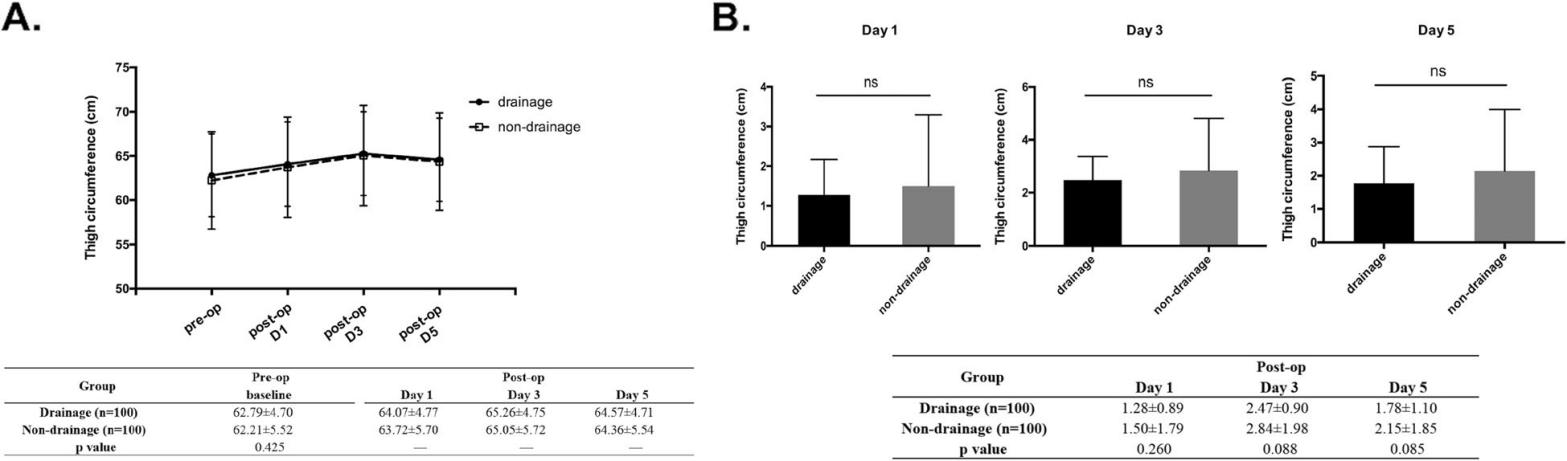
Effect of drainage on thigh circumference after TKA. **a**. the dynamic change of thigh circumference; **b**. increase of the circumference at different time point compared with baseline level

As shown in Fig. 5, the baseline thigh circumference and the increase in the circumference after the operation exhibited no statistically significant differences between the two groups at day 1, 3 and 5.

## Discussion

Wound drainage is commonly used to reduce hematoma formation and the rate of infection in TKA [10]. At surgeons’ discretion, various drainage modes are employed, in terms of time and pressure, among others. However, in recent years, the effect of drainage has been increasingly questioned [11, 12]. *As per* our hypothesis, this retrospective study has shown that, routine placement of CSD, after the administration of tranexamic acid, showed no significant benefit.

### Blood loss and transfusion requirements

Blood loss is a major concern associated with TKA. Excessive bleeding can lead to postoperative anemia, which increases transfusion rates and risk of complications and hampers rehabilitation [13, 14]. As an inhibitor of fibrinolysis, TXA is used to minimize perioperative blood loss and transfusion requirements in patients undergoing TKA. Although some studies suggested that drainage did not increase blood loss, some studies reported the otherwise [15, 16]. These conflicting results might be ascribed to different hemostasis strategies used during the surgery. In our study, blood Hb was taken as an indicator for blood loss, and TXA was intra-articularly injected in all subjects in our series. The results showed that the CSD group experienced more blood Hb loss than those without drainage on postoperative day 1. However, no significant differences were observed at any other time points. The possible explanation might be that it is the self-tamponading of the hematoma that eventually stopped the bleeding process [17]. We believe the bone cutting surface is the major source of bleeding, and the presence of a drainage tube reduces the hemostatic effect. Meanwhile, no transfusion was given in either group according to the criterion that postoperative Hb level drop should be below 70 g/L. We attribute this approximation in the transfusion rate to the limited sample size and the notable hemostatic effect of TXA. Abandoning CSD after TKA, in our opinion, would thus be an advisable choice to decrease blood loss and transfusion rates, and thereby lower the risk of transfusion-associated complications.

### Pain

Postoperative pain is considered to be an adverse factor that impedes recovery. Studies available in literature usually showed that TKAs without drainage might raise soft-tissue tension and cause swelling in the early stage, thereby resulting in more pain. Kim *et al* suggested that using a suction drain would decrease pain by preventing hematoma from forming after TKA [18]. However, Kumar *et al* observed a higher need for opioids in patients receiving CSD might result from the removal of suction drains and discomfort caused by the tube placed in the wound [19]. In our study, no statistically significant differences in VAS score were found between the drainage and non-drainage groups, proving that CSD exerted no impact on postoperative pain. We assum that inflating tourniquet during the surgery can cause nerve and skeletal muscle compression and drastic reperfusion might result upon tourniquet release. Therefore, the prolonged tourniquet application was thought to be a key factor in pain perception after TKA, as demonstrated by previous studies [15]. Moreover, all patients in our study received an intra-articular injection of TXA, which was proved to be an effective method to control bleeding and ease pain to some extent. Our study showed that wound drainage had no benefit in pain relief.

### Function

After recovery from general anesthesia, the isometric contraction of the quadriceps femoris started immediately and the time of active SLR was recorded in each case. As aforementioned, no significant difference in function was observed between the two groups. Similarly, short-term ROM in two groups had no difference either, which was consistent with previous findings [4, 10, 20]. This result indicated that, with blood remaining in the joint cavity instead of being drained, increased intra-articular pressure did not affect ROM. Interestingly, we found that patients who received no drainage had marginally more flexion than those who received drainage at postoperative day 1. This might be attributed to the elimination of the patients’ fear of the drainage and inconvenience of carrying the tube. Moreover, Yin *et al* observed that extension was significantly limited with the presence of a drain during short-term rehabilitation [21]. Nevertheless, long-term follow-up for both flexion and extension ROM revealed no differences.

### Swelling

The limb swelling might be associated with the knee function. Researchers haven’t reached a consensus about whether drainage can ameliorate swelling. In our study, we measured the thigh circumference, 10 cm proximal to the upper border of the patella, to evaluate the degree of the limb swelling. No statistically significant difference in knee circumference was found between the two groups, and the finding was in line with the conclusion arrived at by three previous meta-analyses [12, 16, 22], none of which found any difference between the two groups in knee function and limb swelling. It was believed that limb swelling was mainly caused by the extravasation of blood in the joint cavity to the peripheral tissues. However, the volume of the joint cavity is fixed and can accommodate a limited amount of the blood. The additional drainage could not completely remove the blood in the joint cavity. Furthermore, it is generally believed that the joint cavity bleeding will stop within 12 h after surgery. We observed intriguingly that the degree of swelling in both groups peaked on the third day after surgery. This phenomenon may be due to the protracted inflammatory response in the surrounding tissues caused by resorption of the intra-articular hematoma.

### Complications

Complications in our series were rare (Table 2). Patients in both groups experienced delayed wound healing, whereas no statistically significant difference was found. A variety of factors dictate wound healing, such as age, BMI, nutritional status, suturing, *etc*. Compared with the non-drainage group, drainage entrance, as an additional wound needing suturing, is theoretically at a higher risk of delayed healing. Therefore, we attributed this result to the limited sample size. As to infection, several studies reported that drainage lowered the incidence of postoperative hematoma and, thereby, the incidence of infection [10]. However, some other authors argued that the placement of a drainage tube may increase the infection rate because it provides a portal of entry for bacteria and their retrograde colonization [11, 12]. We found only one knee with drainage developed superficial infection, but no periprosthetic infection was found in our series. Furthermore, no convincing evidence showed that drainage would decrease the incidence of thrombotic events. With early weight-bearing and active practice, no PE or DVT occurred in either group. Since no significant differences in complications existed between the two groups, we were led to believe that it is safe not to use drainage in TKAs.

**Table 2:** Complications

Index	Drainage (*n* = 100)	Non-drainage (*n* = 100)	Total (*n* = 200)	*p* value
Delayed wound healing	5	2	7	0.442
Superficial infection	1	0	1	1.000
Periprosthetic joint infection	0	0	0	–
Deep vein thrombosis	0	0	0	–
Pulmonary embolism	0	0	0	–

### Hospital stay time and cost

Our study found that the hospital stay time was significantly longer in the drainage group than in the non-drainage group. The discharge criteria included freedom from irritation and dry wound and an ROM of flexion/extension 120 °/90 °. All patients satisfied the discharge criteria in this study. Placement of drainage undoubtedly posed a risk of inflammation and prolonged time of blood oozing owing to the additional wound. Therefore, more medication and more dressing changes were needed to meet the discharge criteria, which would delay the discharge in patients receiving drainage. This result was coincident with multiple previous studies [21, 23]. We did not calculate the total expense of treatment and hospitalization in that the physical condition, financial status, coverage of medical insurance and some non-medical factors varied from person to person. However, the prolonged hospital stay time increased the total expense, to some extent.

This study had several limitations. First, it was a retrospective study. Some relevant data, such as the Oxford Knee Score, were not completely collected and were not included in our analyses. Data from prospective randomized studies are more systematic and comprehensive. Second, it was a single-setting study. Results of multiple-center are more convincing. Third, our research lacked long-term clinical and radiographic results. Future follow-up studies focusing on the clinical outcomes are needed. Therefore, the results should be interpreted with caution.

## Conclusions

In conclusion, our study showed that, with the administration of tranexamic acid, the placement of drainage after TKA does not provide more benefits. Our study suggests that a TKA performed without a CSD is safe and feasible after administration of tranexamic acid.

## Data Availability

The datasets used and/or analysed during the current study are available from the corresponding author on reasonable request.

## Abbreviations

CSD: Closed suction drainage
TKA: Total knee arthroplasty
TXA: Tranexamic acid
VAS: Visual analog scale
ROM: Range of motion
SLR: Straight leg raise
